# The mismatch negativity to abstract relationship of tone pairs is independent of attention

**DOI:** 10.1038/s41598-023-37131-y

**Published:** 2023-06-17

**Authors:** Yi-Fang Hsu, Chia-An Tu, Yuchun Chen, Huei-Mei Liu

**Affiliations:** 1 Institute for Research Excellence in Learning Sciences, National Taiwan Normal University, Taipei, 106308 Taiwan; 2 Department of Educational Psychology and Counselling, National Taiwan Normal University, Taipei, 106308 Taiwan; 3 Center of Teacher Education, Fu Jen Catholic University, New Taipei City, 242062 Taiwan; 4 Department of Special Education, National Taiwan Normal University, Taipei, 106308 Taiwan

**Keywords:** Psychology, Human behaviour, Neuroscience, Auditory system, Cognitive neuroscience

## Abstract

The mismatch negativity (MMN) implicating a comparison process between the deviant and the memory trace of the standard can be elicited by not only changes in physical features but also violations of abstract patterns. It is considered pre-attentive, yet the use of the passive design makes it difficult to exclude the possibility of attention leak. In contrast to how this issue has been well addressed with the MMN to physical changes, much less research directly investigated the attentional effect on the MMN to abstract relationships. Here we conducted an electroencephalography (EEG) experiment to study whether and how the MMN to abstract relationships is modulated by attention. We adapted the oddball paradigm of Kujala et al. by presenting occasional descending tone pairs among frequent ascending tone pairs, while additionally implementing a novel control of attention. Participants’ attention was either directed away from the sounds (with an engaging task of visual target detection, so that the sounds were task-irrelevant) or toward the sounds (with a conventional task of auditory deviant detection, so that the sounds were task-relevant). The MMN to abstract relationships appeared regardless of attention, confirming the pre-attentive assumption. The attention-independence of the frontocentral and supratemporal components of the MMN supported the notion that attention is not required to generate the MMN. At the individual level, a relatively equal number of participants showed attention enhancement and attention suppression. It is unlike the attentional modulation on the P3b, which was robustly elicited in the attended condition only. The concurrent collection of these two neurophysiological markers in both unattended and attended conditions might be potentially suitable for testing clinical populations showing heterogeneous deficits in auditory function independent/dependent of attention.

## Introduction

The mismatch negativity (MMN) is an event-related potential (ERP) component to any discriminable change (i.e. deviant) in some repetitive aspect of auditory stimulation (i.e. standard)^1^. Specifically, it is a difference wave between responses to deviant and standard in an oddball paradigm. It peaks at about 100–200 ms after stimulus onset, distributes over frontocentral locations, and is thought to be generated in the auditory cortex (including Heschel’s gyrus (HG) and superior temporal gyrus (STG)) as well as the frontal cortex at a smaller extent with a slight delay^2–4^. It implicates a comparison process between the deviant stimulus and the memory trace of the standard stimulus^5–8^.

Importantly, the comparison process can rest on not only changes in physical features but also violations of abstract rules in the auditory environment^9^. For example, the MMN can be elicited by deviant tone pairs of descending pitches among standard tone pairs of ascending pitches^10–16^. The MMN to abstract relationships therefore suggested that sensory information about two closely spaced stimuli (occurring within the 200–250 ms temporal window of integration) can be integrated into a unitary event to provide a template for the comparison process^15^. Moreover, it showed that the auditory cortex can encode invariant relationships from a set of acoustic variances, supporting the existence of sensory level intelligence^17^. Developmental studies further documented the early ontogenetic ability to extract abstract rules across tones in infants^18^. Since the ability to encode the temporal aspects of sequential auditory information is of essential importance in speech perception, the MMN to abstract relationships was later used to study the auditory function in developmental dyslexia. For example, Kujala et al.^19^ found that, in dyslexic children, audiovisual training enhanced the MMN to occasional descending tone pairs (deviant pairs, *p* = 0.10) among frequent ascending tone pairs (standard pairs, *p* = 0.90) and shortened the reaction time in a discrimination task on deviant versus standard tone pairs. The training effects on the MMN and reaction time were thought to reflect an increased accuracy of auditory representations.

Similar to most studies on the MMN to physical changes, the MMN to abstract relationships was commonly observed in the passive design, where participants read a self-selected book^13–16^ or watched a silent movie^10,11,13^ while ignoring the auditory stimulation. Therefore, it was suggested the processing of abstract attributes also occurs at the pre-attentive level. However, the undemanding nature of the passive design makes it difficult to know to what extent participants followed the instruction to ignore the auditory stimulation (cf. attention leak), leaving it undetermined whether the processing of abstract attributes is indeed independent of attention. The pre-attentive assumption of the MMN to abstract relationships should be explored by identifying the boundary conditions (i.e., the regions of the parameter space in which the theory applies) of its generation. In response to the replication crisis in psychology, it is considered essential to specify the boundary conditions in order to establish a proper derivation chain between test and theory, because a lack of precision about the conditions in which a phenomenon could occur makes it difficult to evaluate empirical discrepancies which either support or oppose a theory^20^. It was further suggested that, to explore boundary conditions, one can either move beyond well-studied conditions to determine whether a phenomenon generalises to the edges of a dimension or to explore regions of parameter space in which a theory might not apply.

One specific approach on this issue is to look into whether and how the MMN to abstract relationships might be modulated by attention. In contrast to how this issue has been well addressed with the MMN to physical changes^21^, much less research directly investigated the attentional effect on the MMN to abstract relationships. Using magnetoencephalography (MEG), Pardo and Sams^22^ examined the MMN to rising and falling glides in unattended and attended conditions. Participants read a self-selected book in the unattended condition (as in the passive design) and counted the deviant in the attended condition. They reported the MMN in both conditions, where attention did not affect its magnitude. In a similar vein, Van Zuijen et al.^23^ used electroencephalography (EEG) to examine the MMN to rising and falling tone pairs in unattended and attended conditions. Participants watched a silent movie in the unattended condition (as in the passive design) and pressed a button to the deviants in the attended condition. They found significant MMN in both conditions. Although the MMN appeared smaller in unattended than attended condition, no statistical comparison was made between the two attention levels. On the other hand, Paavilainen et al.^24^ adopted a strict control of attention, using a dichotic listening task to record the MMN to rising and falling tone pairs in the unattended ear (where participants ignored the sounds) and the attended ear (where participants pressed a button to the deviants). They documented the MMN in both unattended condition (on the right but not the left ear) and attended condition (in hits but not misses), which appeared smaller in the former than the latter yet no statistical comparison was made between the two attention levels. Moreover, the pattern of the MMN was more ambiguous in comparison to that obtained with the aforementioned passive design, raising the possibility that the representation of acoustic pattern might place more demands on attentional resources^25^ thus cannot be a purely automatic process. This idea was supported by Tervaniemi et al.^26^ using melodic contours of five tones as stimuli. Participants were classified into “accurate” and “inaccurate” groups depending on how well they identified the deviant versus standard melodic contours in the attended condition. In the unattended condition, the MMN was absent in both groups at the first phase of the experiment and then appeared in “accurate” but not “inaccurate” participants at later phase of the experiment. It was suggested that the formation of representations for complicated patterns needed some attentive listening. Only after the representations had emerged, the pre-attentive detection of abstract relationships can occur. Altogether, it remains undetermined whether and how the MMN to abstract relationships might be modulated by attention.

In order to examine the pre-attentive assumption of the MMN to abstract relationships by identifying the boundary conditions of its generation, here we adapted the oddball paradigm of Kujala et al.^19^ where participants were presented with occasional descending tone pairs (750–500 Hz deviant pairs, *p* = 0.10) among frequent ascending tone pairs (500–750 Hz standard pairs, *p* = 0.90). Specifically, we replaced two tones with eight tones as stimuli to introduce variation in pitch, so that the distinction between deviant pairs and standard pairs did not lie in order reversals but depend on the extraction of a relational rule. We additionally implemented a novel control of attention, where participants were instructed to perform an engaging task of visual target detection (i.e. unattended condition) or a conventional task of auditory deviant detection (i.e. attended condition), which was reported to successfully direct participants’ attention away from or toward the auditory stimulation^27^. These arrangements created boundary conditions for the elicitation of a robust MMN to abstract relationships. We hypothesised that, if the processing of acoustic pattern requires at least some amount of attention, the MMN should be absent in the unattended condition. In contrast, if the processing of acoustic pattern indeed occurs at the pre-attentive level, the MMN should be present in the unattended condition.

## Materials and methods

### Participants

A total of 20 healthy volunteers participated in the study (age mean (SD) = 21.15 (0.67), 12 males, 19 right-handed), reporting no history of neurological, neuropsychiatric, or visual/hearing impairments. All participants gave written informed consent and were paid for participation. A power analysis was conducted in G ∗ Power 3.1.9.7^28,29^ using a model for paired samples t-tests with a sample size of 20, which was larger than or comparable to the sample size of previous studies on attentional modulation of the abstract MMN (N = 6 in Ref.^22^; N = 9 in Ref.^24^; N = 11*2 in Ref.^19^; N = 20 in Ref.^26^; N = 23 in Ref.^23^). With alpha set at 0.05, the power to detect a small-sized effect (0.20) was 0.14, the power to detect a medium-sized effect (0.50) was 0.56, and the power to detect a large-sized effect (0.80) was 0.92. The study was conducted in accordance with the Declaration of Helsinki and approved by the Research Ethics Committee at National Taiwan Normal University.

### Stimuli

Participants were presented with sequences of tone pairs with a constant pair-onset asynchrony of 770 ms. Each tone was 50 ms in duration (including 5 ms rise/fall times), generated in Sound Forge Pro 10.0 (Sony Creative Software Inc.). There were eight tones within the range of 493.88–987.77 Hz, matching the absolute frequency of a series of eight natural keys on a modern piano (i.e., B4 C5 D5 E5 F5 G5 A5 B5). The standard pair, which occurred in 90% of the trials, was ascending in pitch to the next higher tone (e.g. F5-G5). The deviant pair, which occurred randomly in 10% of the trials, was descending in pitch to the next lower tone (e.g. E5-D5). The two tones within a pair were separated by a 150-ms silent gap. In other words, the duration of a tone pair was 250 ms, which remained within the temporal window of integration^15^.

### Procedures

Participants were presented with 4 unattended blocks (containing 800 tone pairs) followed by 4 attended blocks (containing 800 tone pairs). Auditory stimulation was delivered binaurally via headphones (Sennheiser HD 2.30, with an intensity of 65.4–72.3 dBA (64.4–71.1 dBC)). Each block started with at least 10 standard pairs before a deviant pair appeared. Participants were seated in front of a computer screen viewed from a distance of 120 cm. In the unattended blocks, participants were asked to ignore the auditory stimulation and count the number of shots in a silent National Basketball Association (NBA) highlight clip (spanning 2 blocks, containing 43 shots) and the number of characters in a silent Moomin animation clip (spanning 2 blocks, containing 12 characters) and report it at the end of each clip. The NBA highlight clip could engage participants’ attention because participants had to follow the fast tempo of the game. The Moomin animation clip could engage participants’ attention because participants had to track a range of unfamiliar but similar characters coming in and out of the scene. Each clip (fast-forwarded from the original, lasting 6 min 30 s) started before the first auditory stimulation and ended after the last auditory stimulation. In the attended blocks, participants were instructed to count the number of deviant pairs (which occurred 18–22 times per block) and report it at the end of each block. These tasks were reported to successfully direct participants’ attention away from or toward the auditory stimulation^27^. Note that in both unattended and attended conditions, participants performed the visual target detection and auditory deviant detection by reporting the detected number at the end of each block, rather than by pressing a button upon detection as the experiment went on. This was to minimise motor-related artefacts, which came with the cost of not being able to monitor participants’ behavioural performance online. A fixation cross shown in grey against black background was displayed on the screen. E-prime version 2.0 (Psychology Software Tools) was used for stimulus presentation.

### Data recording and analysis

#### EEG recording and pre-processing

EEG was recorded from 32 active electrodes on a Brain Products actiCAP snap according to the extended 10–20 system. The ground electrode was placed at FPz and the reference electrode was placed at Fz. Eye movements were monitored by additional four electrodes placed above and below the left eye and at the outer canthi of both eyes, which were bipolarized online to yield vertical and horizontal electrooculography (EOG), respectively. All signals were amplified with the BrainVision actiCHamp Plus (Brain Products GmbH, Germany) and sampled at 500 Hz, and then filtered at 0.1–100 Hz offline.

Ocular artefact correction was conducted with independent component analysis (ICA) in EEGlab 14_1_2b^30^ using the runica algorithm. Independent components capturing blinks and horizontal eye movements were determined by a criterion of at least 70% confidence in the eye category, pruning out 1 to 4 components for each participant.

Epochs extended from − 100 ms to 700 ms relative to the onset of tone pair, using a − 100 ms to 0 ms pre-stimulus baseline. Bad electrodes were identified (if there were more than 25% of the epochs containing voltage deviations exceeding ± 100 μV relative to baseline) and interpolated using spherical interpolation. The data was recomputed to average reference. Epochs containing voltage deviations exceeding ± 100 μV relative to baseline at any of the electrodes were rejected. Lastly, the data was lowpass-filtered at 30 Hz. The trial numbers after artefact rejection in each condition are listed in Table 1.

**Table 1:** Range, mean, and SD of trial numbers after artefact rejection in each condition.

	Unattended		Attended	
	Standard	Deviant	Standard	Deviant
Range	57–80	55–80	63–80	59–80
Mean	77.20	76.45	77.00	76.75
SD	5.44	6.00	5.34	5.80

#### ERP analysis

To obtain the neural activity associated with the discrimination of standard and deviant, we calculated the difference waves by subtracting the ERPs of the standard pair (immediately prior to the deviant pair) from the ERPs of the deviant pair. Note that equal number of trials were selected for standard and deviant to ensure that the signal-to-noise ratio are similar between conditions.

The difference waves were submitted to a temporal principal component analysis (PCA) in SPSS 23. Since it was first introduced^31,32^, PCA has been considered an effective linear reduction method for multivariate ERP data^33–41^. It statistically decomposes the ERP waveforms into constituent building blocks, which affords data-driven ERP component measures compared with other conventional methods^40,42,43^. Moreover, it is not as susceptible to the influences of high-frequency noises and low-frequency drifts in the data as other conventional methods^44^. The data used for component extraction included data from all electrodes and all conditions of each participant. Covariance matrix and Promax rotation were used here. All components accounting for a total of 99% of the variance (maximum iterations for convergence = 500) were included in the rotation (Promax kappa = 4). The decomposition provided a set of time-variant component loadings reflecting the contribution of each temporal component to the voltage at each time point and a set of time-invariant component scores (calculated using Bartlett method) representing the contribution of each temporal component to the ERP waveforms which can be subject to inferential statistics^45^.

We identified PCs corresponding to the MMN and the P3b on the basis of the component loading latencies and the component score topographies. Specifically, we identified from 48 PCs one PC at around 350 ms corresponding to the MMN (i.e., PC2 accounting for 10.73% of the variance) and one PC at around 600 ms corresponding to the P3b (i.e. PC1 accounting for 47.03% of the variance). The component scores were averaged across three electrodes showing the most negative/positive responses across all conditions independent of experimental manipulation to serve as objective representatives of the components. The advantage of averaging three maximum electrodes was twofold. First, it increased the signal-to-noise ratio of the components. Second, it avoided the problems inherited in the analysis of predefined areas that took an average of multiple electrodes over pre-defined regions, which might not correspond to the true topography in the experiment. Therefore, we performed paired samples t-tests on the averages of these electrodes to test for the effect of attention.

## Results

### Behavioural data

In the unattended condition, participants were asked to ignore the auditory stimulation and count the number of shots in a silent NBA highlight clip and the number of characters in a silent Moomin animation clip. In the attended condition, participants were instructed to count the number of deviant pairs. Table 2 lists the mean and SD of reported targets in unattended and attended conditions. The performance was close to ceiling, suggesting that participants followed the instruction to shift their attention.

**Table 2:** Mean and SD of reported targets in unattended and attended conditions.

	Unattended		Attended			
	NBA	Moomin				
	Block 1 + 2	Block 3 + 4	Block 5	Block 6	Block 7	Block 8
Actual targets	43.00	12.00	20.00	18.00	22.00	20.00
Mean	42.45	11.80	19.40	16.55	17.35	17.60
SD	1.54	0.52	8.06	8.64	7.70	8.62

### ERP data

**Figure 1:**
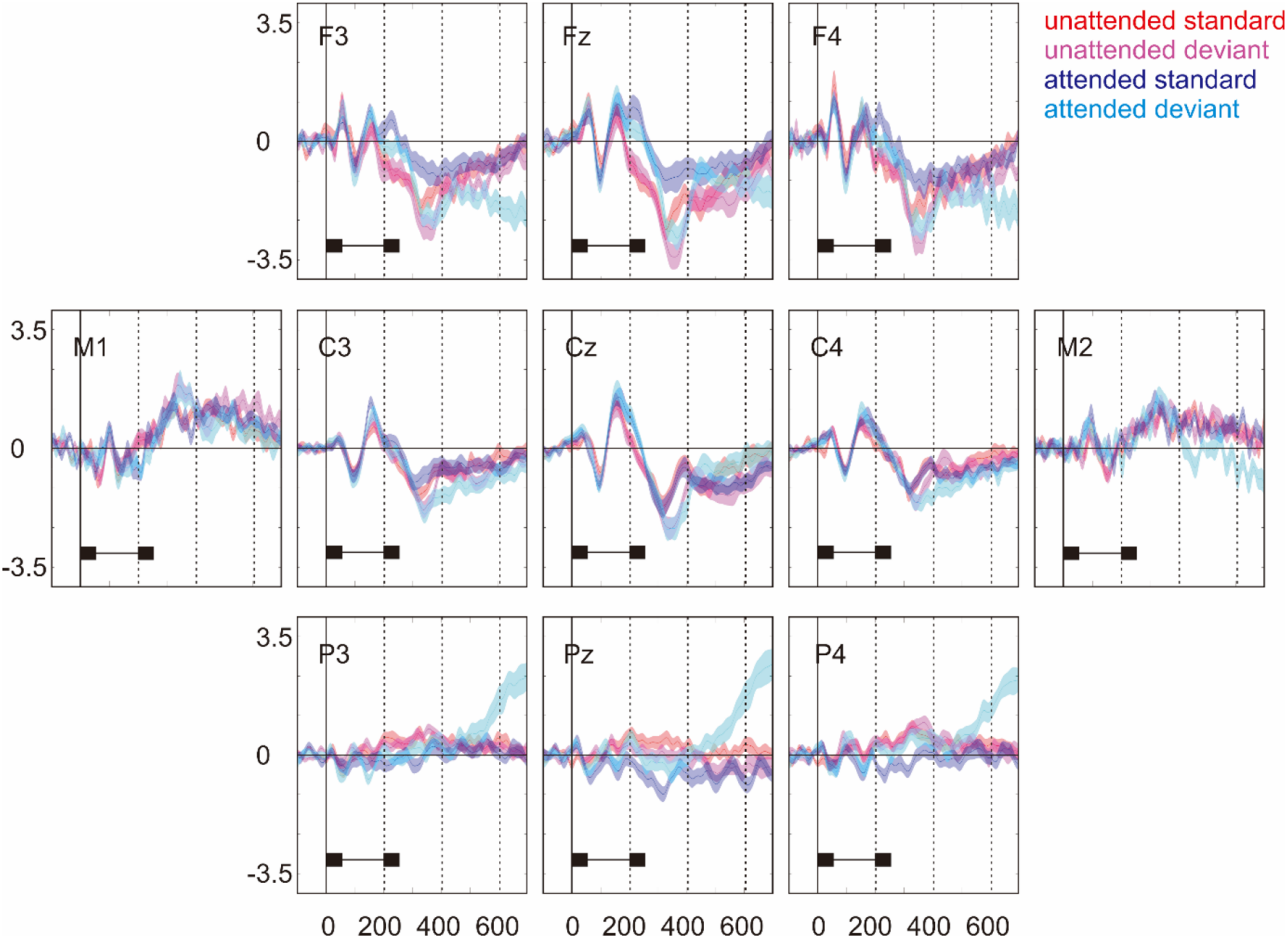
The ERPs to standard and deviant on nine representative electrodes as well as left and right mastoids (i.e. M1 and M2). Shaded area represents the standard error of the mean. The temporal course of a tone pair is shown below the ERPs.

**Figure 2:**
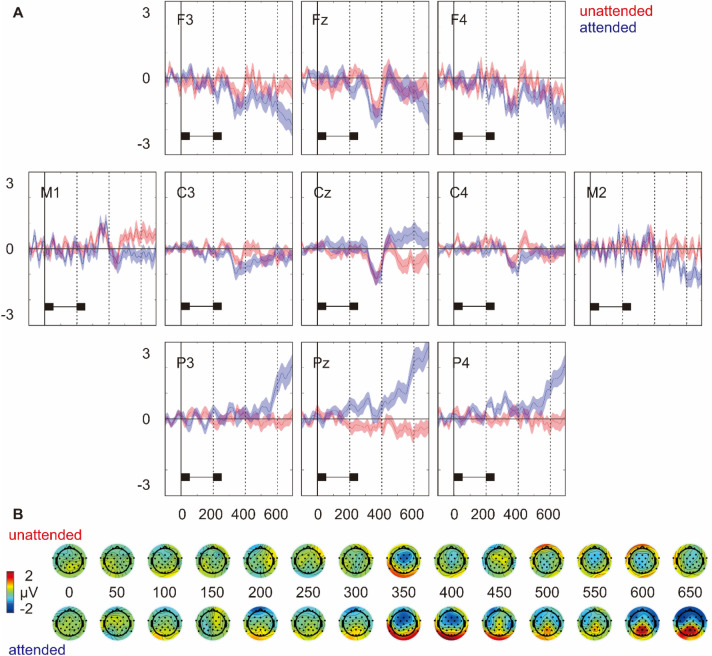
(**A**) The difference waves (i.e. deviant—standard) on nine representative electrodes as well as left and right mastoids (i.e. M1 and M2). Shaded area represents the standard error of the mean. The temporal course of a tone pair is shown below the ERPs. (**B**) Topographical distributions of the difference waves plotted from 0 to 650 ms in 50 ms steps after the onset of a tone pair.

Figure 1 illustrates the ERPs to standard and deviant. Figure 2 illustrates the difference waves (i.e. deviant—standard) and the topographical distributions of the difference waves. The MMN (emerging at around 350 ms) can be seen in both unattended and attended conditions, showing a frontocentral distribution and a polarity reversal at the mastoids. On the other hand, the P3b (emerging at around 600 ms) was absent in the unattended condition but present in the attended condition, as a parietal positivity was elicited by attended deviant relative to attended standard.

**Figure 3:**
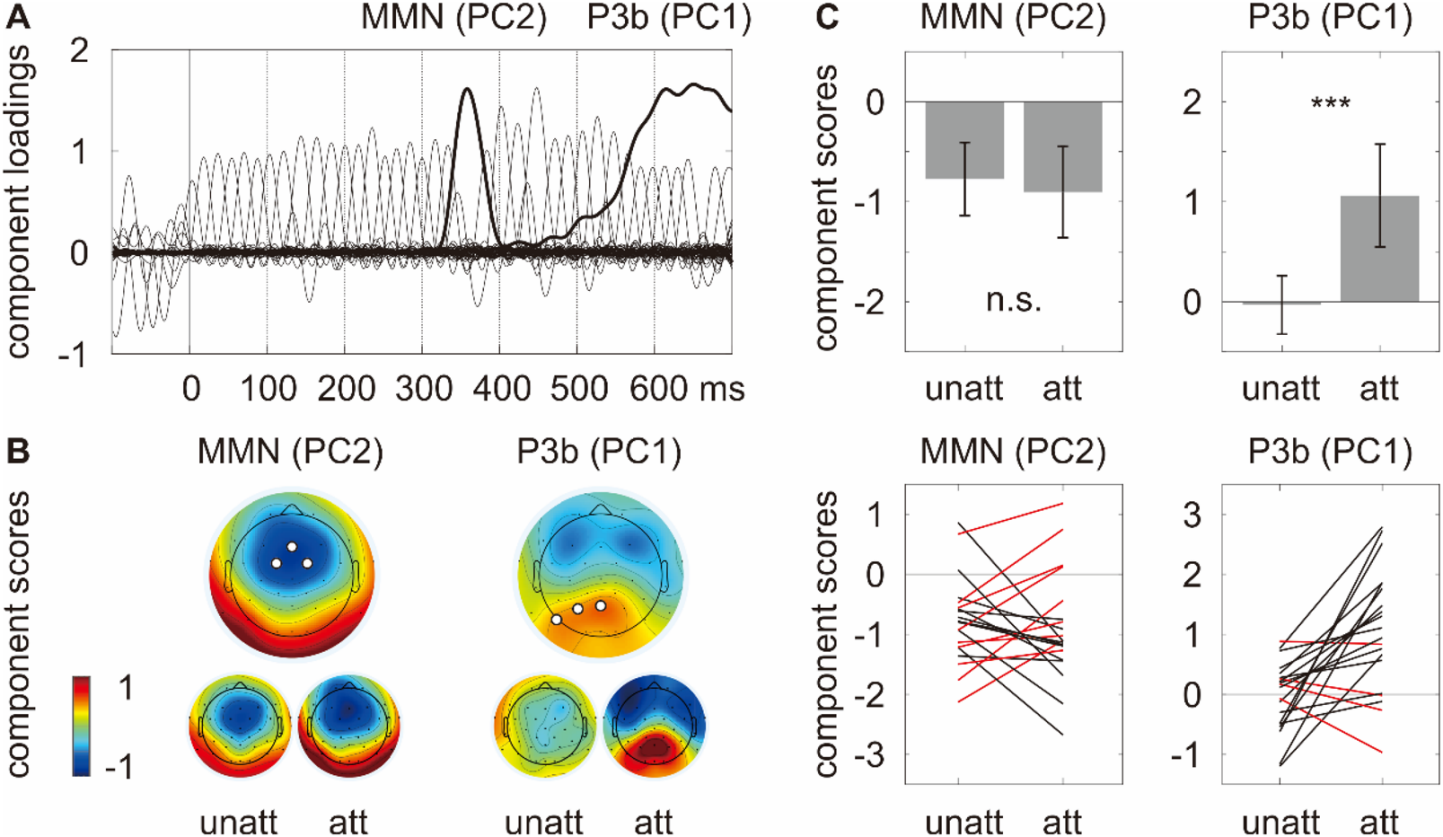
(**A**) Component loadings of the PCs. The PCs corresponding to the MMN and the P3b are marked with thick lines. (**B**) The component score topographies, where three electrodes showing the most negative/positive responses independent of experimental manipulation are marked as white dots. (**C**) Component scores averaged across three maximum electrodes in unattended and attended conditions at the group level (upper, where the error bar depicts one standard deviation of the mean) and the individual level (lower, where participants showing the opposite pattern to the average are marked in red).

The MMN and the P3b were respectively identified using the temporal PCA (Fig. 3A). Figure 3B shows the component score topographies. Figure 3C shows the component scores averaged across three maximum electrodes in unattended and attended conditions at the group level and the individual level, which were submitted to paired samples t-tests to test for the effect of attention.

**Figure 4:**
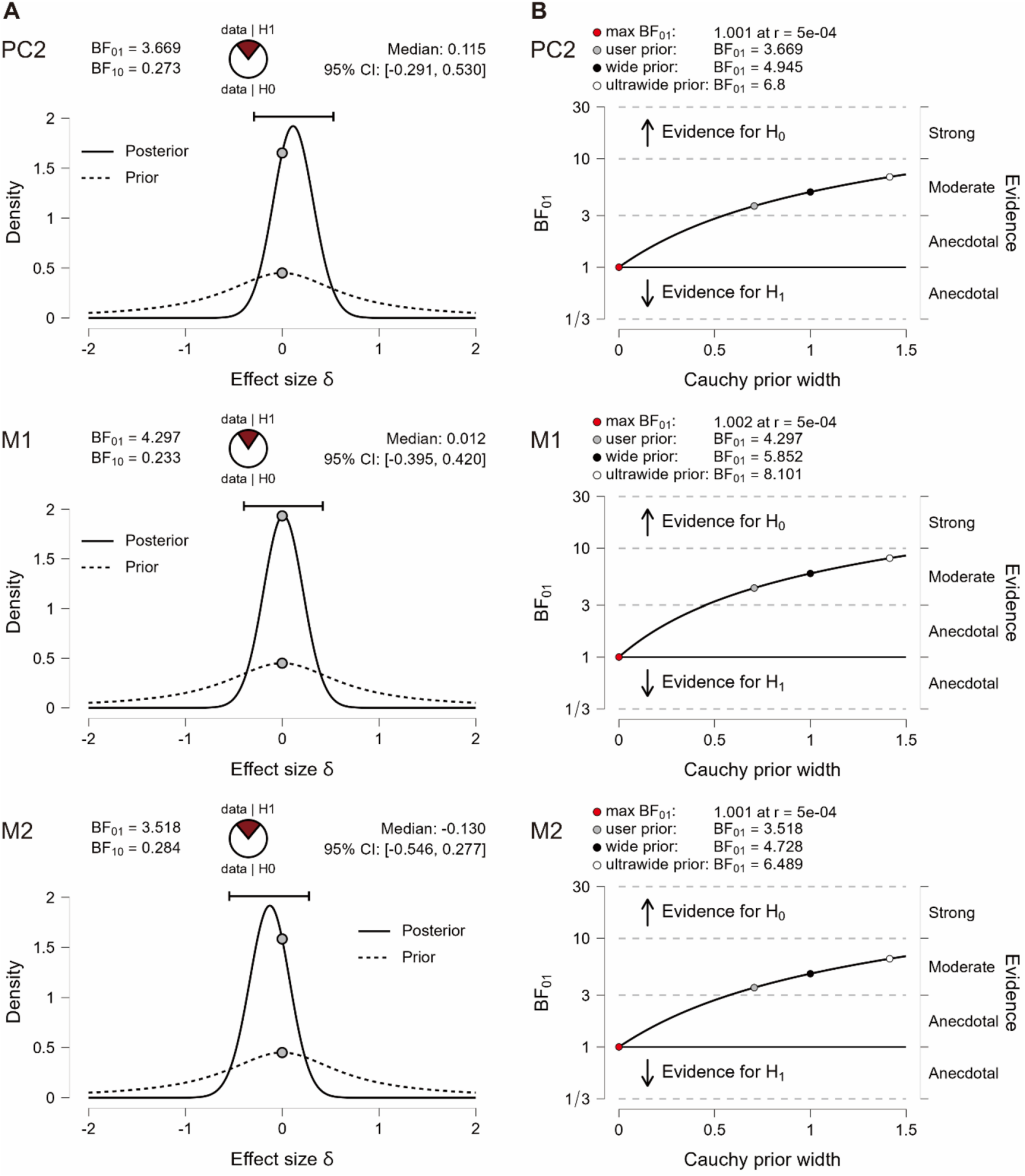
(**A**) Bayesian paired samples t-tests for the parameter δ. (**B**) The Bayes factor robustness plot. The maximum BF_01_ is attained when setting the prior width r to 5e − 04.

Paired samples t-tests showed that the MMN (identified as PC2) was not significantly modulated by attention (*t*(19) = 0.60, *p* = 0.56). While 11/20 participants showed an attention enhancement, 9/20 participants showed an attention suppression (Fig. 3C left). Meanwhile, its polarity reversal at the mastoids was not significantly modulated by attention (M1: *t*(19) = 0.06, *p* = 0.95; M2: *t*(19) = − 0.67, *p* = 0.51). The evidence of absence was supported by the results of Bayesian paired samples *t*-tests in JASP Version 0.17.1.0 (JASP Team, 2023), showing that the null hypothesis predicts the data 3.52–4.30 times better than the alternative hypothesis on the aforementioned three indices (Table 3 and Fig. 4). On the other hand, the P3b (identified as PC1) was absent in the unattended condition but present in the attended condition (*t*(19) = − 4.02, *p* ≤ 0.001). This pattern can be seen in 16/20 participants (Fig. 3C right).

**Table 3:** Bayesian paired samples t-tests on the MMN (identified as PC2) and its polarity reversal at M1 and M2.

MMN	BF_01_	Error %
PC2	3.67	0.02
M1	4.30	0.02
M2	3.52	0.02

**Figure 5:**
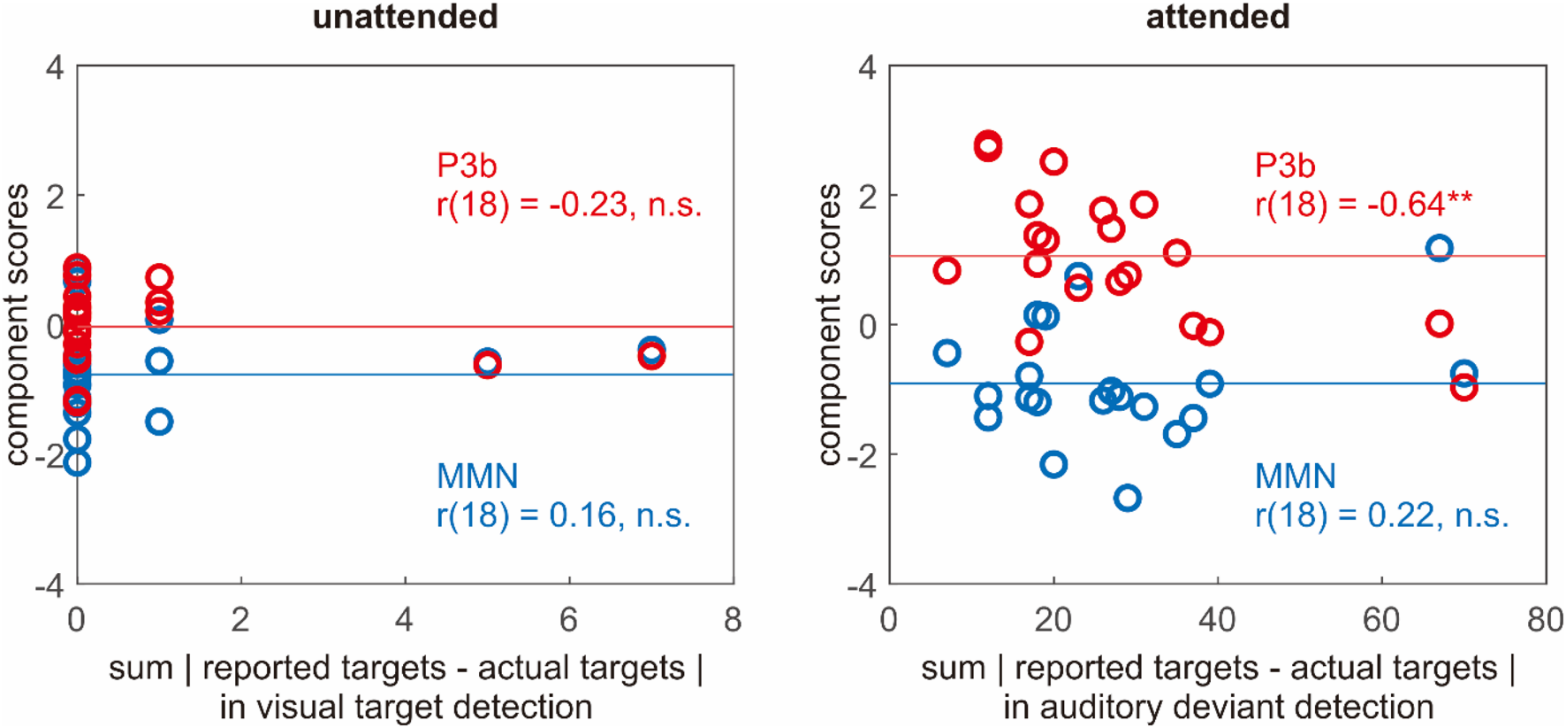
Scatter plots showing the relationship between participants’ performance on the behavioural tasks (x-axis, quantified as how much the reported targets deviated from the actual targets) and the size of the MMN and the P3b (y-axis, quantified as component scores averaged across three maximum electrodes). The horizontal lines mark the mean of the MMN and the P3b, respectively in blue and red.

In order to explore how individual differences in performing the behavioural tasks might have contributed to the effect of attention on the ERPs, we examined the relationship between participants’ performance on the behavioural tasks and the size of the MMN and the P3b. In the unattended condition (Fig. 5 left), participants’ performance on visual target detection did not correlate with the size of the MMN and the P3b (MMN: *r*(18) = 0.16, *p* = 0.51; P3b: *r*(18) = − 0.23, *p* = 0.33). Specifically, for the 5/20 participants whose performance was not at ceiling (who might be suspected of having attention leak), the size of the MMN and the P3b did not seem to digress from the rest of the participants. In other words, although some participants did not excel at visual target detection, there was little evidence that they attended to the sounds instead. In the attended condition (Fig. 5 right), participants’ performance on auditory deviant detection did not correlate with the size of the MMN (*r*(18) = 0.22, *p* = 0.35), while participants better at auditory deviant detection did exhibit larger P3b (*r*(18) = − 0.64, *p* < 0.01).

## Discussion

The MMN elicited by deviant tone pairs among standard tone pairs indicated that sensory information about two closely spaced stimuli can be integrated into a unitary event^15^ and more importantly, that the auditory cortex is able to encode invariant relationships from a set of acoustic variances^17^. Since the MMN to abstract relationships was commonly observed in the passive design, previous studies proposed a pre-attentive discrimination process of abstract attributes in the auditory system. Nevertheless, the undemanding nature of the passive design makes it difficult to exclude the possibility of attention leak, yet much less research directly investigated the attentional effect on the MMN to abstract relationships^22–24,26^. Here we examined the pre-attentive assumption by studying whether and how the MMN to abstract relationships might be modulated by attention. We adapted the oddball paradigm of Kujala et al.^19^ while additionally implementing a novel control of attention. Participants’ attention was either directed away from the auditory stimulation with an engaging task of visual target detection (i.e., unattended condition) or toward the auditory stimulation by a conventional task of auditory deviant detection (i.e., attended condition)^27^. Such manipulation was adopted for the following reasons. First, it is subjectively appealing for today’s participants to focus on rare events in short video clips. Second, this task provides simple, objective, and quantitative measures of participants’ performance on the cover task. We found the MMN regardless of attention, confirming that the processing of abstract attributes can occur at the pre-attentive level^10–16^. This result supported the notion that attention is not required to generate the MMN^25,46^.

### The MMN appeared regardless of attention

The MMN in the unattended condition lent straightforward support to the pre-attentive assumption of the MMN to abstract relationships. It was observed when participants’ attention was directed away from the auditory stimulation with an engaging task of visual target detection rather than when participants read a self-selected book or watched a silent movie^22,23^, so that the possibility of attention leak was minimal. This is in line with previous studies on anesthetised rodents showing that the rapid representation of abstract rule is not restricted to the awake state of the brain^47^. The MMN in the attended condition, on the other hand, can be difficult to disentangle from the temporally overlapping components such as the P165 and the N2b^6,8^. Nevertheless, the presence of the frontocentral negativity with a polarity reversal at the mastoids suggests that it can be identified as the MMN rather than other components. Altogether, it seems that the MMN signalling a discrimination process of complicated regularities can occur regardless of attention.

In the current research, we adapted the oddball paradigm of Kujala et al.^19^ by replacing two tones with eight tones as stimuli to introduce variation in pitch, ensuring that the distinction between deviant pairs and standard pairs did not lie in order reversals but depend on the extraction of a relational rule. In this case, pattern violation can be detected only after a tone pair ended. Indeed, the MMN in the current research emerged at around 350 ms. With the duration of a tone pair being 250 ms, it fell right within the typical MMN time window of 100 ms after a tone pair ended. This provided unequivocal evidence that the observed response cannot be the MMN to physical changes (in the first tone of a tone pair) but the MMN to abstract violations (of a tone pair), signalling the processing of invariant relationships from a set of acoustic variances.

Interestingly, previous studies on the MMN to physical changes reported that attention could enhance the MMN to hard-to-detect deviants but not easy-to-detect deviants^48–50^. It was proposed that the more salient the deviants, the more likely it can trigger an involuntary switch of attention, overwriting the effect of task-relevance. The lack of attentional effect in the current research suggested that, even when the deviant-standard distinction lied in the extraction of a relational rule, the discrimination of occasional descending tone pairs among frequent ascending tone pairs might still be an easy task for the auditory system.

### Attention modulated the P3b

Following the MMN, we also observed the P3b which was modulated by attention. The PC corresponding to the P3b was absent in the unattended condition but present in the attended condition. In the literature, the P3b is thought to involve a brain-scale cortical network including prefrontal, parietal, temporal, and cingulate regions^51,52^, signalling contextual updating in working memory^53–55^. Previous studies on the hierarchical processing of auditory regularities also showed that the P3b is highly dependent on conscious awareness of stimulus regularity^56^. Specifically, the P3b to violation of global regularities decreased when participants engaged in mind-wandering and disappeared when participants engaged in a cover task of visual target detection. Our results confirmed that the P3b can serve as a marker of attention, indexing conscious awareness of auditory regularities.

### The effective manipulation of attention

Here we implemented a novel control of attention. Participants’ attention was either directed away from the sounds (with an engaging task of visual target detection, hence the unattended condition where sounds were task-irrelevant) or toward the sounds (with a conventional task of auditory deviant detection, hence the attended condition where sounds were task-relevant). Indeed, previous studies on the MMN to physical changes offered a range of alternatives to manipulate participants’ attention, where different types of visual tasks were employed to examine the cross-modal effect of attentional load^57^. While the current research made a novel attempt, a crucial question is whether the manipulation of attention was effective, that is, whether it managed to differentiate between the unattended and attended conditions. Could it be that attention was involved when participants should be counting the visual targets (in the unattended condition) or that attention was not involved when participants should be counting the auditory deviants (in the attended condition)? We consider these scenarios unlikely because the behavioural data showed that participants’ performance was close to ceiling (Table 2) and more importantly, the ERPs data showed that the P3b was absent in the unattended condition but present in the attended condition (Figs. 1 and 2). While we cannot unequivocally determine whether the close-to-ceiling performance on the behavioural tasks might result from participants’ engaging in the tasks or the tasks being too simple, the respective absence/presence of the P3b in the unattended/attended condition suggested that participants did not attend to the sounds in the same way in the two conditions.

### Limitations

The experimental design of the current research is subject to the following limitations. First, here the unattended blocks always preceded the attended blocks. This was arranged to prevent the unattended condition from being contaminated by the identification of the sounds in the attended condition^58^, which might dampen the effectiveness of the attention manipulation. However, the fixed order of block might also introduce a confounding effect of fatigue, practice, or learning which was reported to attenuate the MMN to physical changes^59–61^. Future studies might consider counterbalancing the order of blocks to explore this possibility. Second, here the ascending tone pairs always served as standards and the descending tone pairs always served as deviants, following the experimental design of previous studies on the MMN to abstract relationships^10–16,19^. While similar findings of the MMN to abstract relationships were documented with different directions of change^62,63^, it is unknown whether the reversed assignment of ascending and descending tone pairs might interact with our manipulation of attention. Future studies might consider counterbalancing the directions of change to explore this possibility.

## Conclusion

Overall, the current research showed that the MMN to abstract relationships is a pre-attentive component, as it appeared regardless of attention. It is in contrast to the attentional modulation on the P3b, which was elicited in the attended condition only. Notably, in the current research, the attention-independent MMN seemed to result from a relatively equal number of participants showing attention enhancement and attention suppression, whereas the attentional effect on the P3b was quite robust even at the individual level. The concurrent collection of these two neurophysiological markers in both unattended and attended conditions might be potentially suitable for testing clinical populations showing heterogeneous deficits in auditory function independent/dependent of attention.

## References

[1] Kujala T, Lovio R, Lepistö T, Laasonen M, Näätänen R (2006). Evaluation of multi-attribute auditory discrimination in dyslexia with the mismatch negativity. Clin. Neurophysiol..

[2] Chennu S (2016). Silent Expectations: Dynamic causal modeling of cortical prediction and attention to sounds that weren’t. J. Neurosci..

[3] Rinne T, Alho K, Ilmoniemi RJ, Virtanen J, Näätänen R (2000). Separate time behaviors of the temporal and frontal mismatch negativity sources. Neuroimage.

[4] Alho K (1995). Cerebral generators of mismatch negativity (MMN) and its magnetic counterpart (MMNm) elicited by sound changes. Ear Hear..

[5] Schröger E (1998). Measurement and interpretation of the mismatch negativity. Behav. Res. Methods Instrum. Comput..

[6] Näätänen R (2000). Mismatch negativity (MMN): Perspectives for application. Int. J. Psychophysiol..

[7] Näätänen R (2003). Mismatch negativity: Clinical research and possible applications. Int. J. Psychophysiol..

[8] Näätänen R, Escera C (2000). Mismatch negativity: Clinical and other applications. Audiol. Neurotol..

[9] Paavilainen P (2013). The mismatch-negativity (MMN) component of the auditory event-related potential to violations of abstract regularities: A review. Int. J. Psychophysiol..

[10] Gumenyuk V (2003). Electric brain responses indicate preattentive processing of abstract acoustic regularities in children. NeuroReport.

[11] Korzyukov OA, Winkler I, Gumenyuk VI, Alho K (2003). Processing abstract auditory features in the human auditory cortex. Neuroimage.

[12] Paavilainen P, Jaramillo M, Näätänen R (1998). Binaural information can converge in abstract memory traces. Psychophysiology.

[13] Paavilainen P, Jaramillo M, Näätänen R, Winkler L (1999). Neuronal populations in the human brain extracting invariant relationships from acoustic variance. Neurosci. Lett..

[14] Saarinen J, Paavilainen P, Schröger E, Tervaniemi M, Näätänen R (1992). Representation of adstract attributes of auditory stimuli in human brain. NeuroReport.

[15] Tervaniemi M, Saarinen J, Paavilainen P, Danilova N, Näätänen R (1994). Temporal integration of auditory information in sensory memory as reflected by the mismatch negativity. Biol. Psychol..

[16] Tervaniemi M, Radil T, Radilova J, Kujala T, Näätänen R (1999). Pre-attentive discriminability of sound order as a function of tone duration and interstimulus interval: A mismatch negativity study. Audiol. Neurotol..

[17] Näätänen R, Tervaniemi M, Sussman E, Paavilainen P, Winkler I (2001). ‘Primitive intelligence’ in auditory cortex. Trends Neurosci..

[18] Carral V, Huotilainen M, Ruusuvirta T, Fellman V, Näätänen R, Escera C (2005). A kind of auditory ‘primitive intelligence’ already present at birth. Eur. J. Neurosci..

[19] Kujala T (2001). Plastic neural changes and reading improvement caused by audiovisual training in reading-impaired children. Proc. Natl. Acad. Sci. U. S. A..

[20] Scheel AM, Tiokhin L, Isager PM, Lakens D (2021). Why hypothesis testers should spend less time testing hypotheses. Perspect. Psychol. Sci..

[21] Sussman ES (2007). A new view on the MMN and attention debate: The role of context in processing auditory events. J. Psychophysiol..

[22] Pardo PJ, Sams M (1993). Human auditory cortex responses to rising versus falling glides. Neurosci. Lett..

[23] Zuijen TLV, Simoens VL, Paavilainen P, Näätänen R, Tervaniemi M (2006). Implicit, intuitive, and explicit knowledge of abstract regularities in a sound sequence: An event-related brain potential study. J. Cogn. Neurosci..

[24] Paavilainen P, Saarinen J, Tervaniemi M, Näätänen R (1995). Mismatch negativity to changes in abstract sound features during dichotic listening. J. Psychophysiol..

[25] Alain C, Woods DL (1997). Attention modulates auditory pattern memory as indexed by event-related brain potentials. Psychophysiology.

[26] Tervaniemi M, Rytkönen M, Schröger E, Ilmoniemi RJ, Näätänen R (2001). Superior formation of cortical memory traces for melodic patterns in musicians. Learn. Mem..

[27] Hsu Y-F, Hämäläinen JA (2021). Both contextual regularity and selective attention affect the reduction of precision-weighted prediction errors but in distinct manners. Psychophysiology.

[28] Faul F, Erdfelder E, Lang A-G, Buchner A (2007). G*Power 3: A flexible statistical power analysis program for the social, behavioral, and biomedical sciences. Behav. Res. Methods.

[29] Faul F, Erdfelder E, Buchner A, Lang A-G (2009). Statistical power analyses using G*Power 3.1: Tests for correlation and regression analyses. Behav. Res. Methods.

[30] Delorme A, Makeig S (2004). EEGLAB: An open source toolbox for analysis of single-trial EEG dynamics including independent component analysis. J. Neurosci. Methods.

[31] Donchin E (1966). A multivariate approach to the analysis of average evoked potentials. IEEE. Trans. Biomed. Eng.

[32] Ruchkin DS, Villegas J, John ER (1964). An analysis of average evoked potentials making use of least mean square techniques. Ann. N. Y. Acad. Sci..

[33] Chapman RM, Mccrary JW (1995). EP component identification and measurement by principal components-analysis. Brain Cogn..

[34] Dien J (1998). Addressing misallocation of variance in principal components analysis of event-related potentials. Brain Topogr..

[35] Dien J., & Frishkoff G.A., Principal components analysis of event-related potential datasets. *Event-Relat. Potentials Methods Handb.* 189–208 (2005).

[36] Duffy FH, Jones K, Bartels P, McAnulty G, Albert M (1992). Unrestricted principal components analysis of brain electrical activity: Issues of data dimensionality, artifact, and utility. Brain Topogr..

[37] Möcks J (1988). Decomposing event-related potentials: A new topographic components model. Biol. Psychol..

[38] Möcks J, Gasser T, Köhler W (1988). Basic statistical parameters of event-related potentials. J. Psychophysiol..

[39] Picton TW, Bentin S, Berg P, Donchin E, Hillyard SA, Johnson R, Miller GA, Ritter W, Ruchkin DS, Rugg MD, Taylor MJ (2000). Guidelines for using human event-related potentials to study cognition: Recording standards and publication criteria. Psychophysiology.

[40] Kayser J, Tenke CE (2003). Optimizing PCA methodology for ERP component identification and measurement: Theoretical rationale and empirical evaluation. Clin. Neurophysiol..

[41] Dien J (2012). Applying principal components analysis to event-related potentials: A tutorial. Dev. Neuropsychol..

[42] Beauducel A, Debener S, Brocke B, Kayser J (2000). On the reliability of augmenting/reducing: Peak amplitudes and principal component analysis of auditory evoked potentials. J. Psychophysiol..

[43] Kayser J, Tenke CE, Bruder GE (1998). Dissociation of brain ERP topographies for tonal and phonetic oddball tasks. Psychophysiology.

[44] Luck SJ (2005). An Introduction to the Event-Related Potential Technique.

[45] Van Boxtel GJM (1998). Computational and statistical methods for analyzing event-related potential data. Behav. Res. Methods Instrum. Comput..

[46] Näätänen R, Paavilainen P, Tiitinen H, Jiang D, Alho K (1993). Attention and mismatch negativity. Psychophysiology.

[47] Astikainen P, Ruusuvirta T, Näätänen R (2014). Rapid categorization of sound objects in anesthetized rats as indexed by the electrophysiological mismatch response. Psychophysiology.

[48] Alho K, Woods DL, Algazi A, Näätänen R (1992). Intermodal selective attention. II. Effects of attentional load on processing of auditory and visual stimuli in central space. Electroencephalogr. Clin. Neurophysiol..

[49] Woods DL (1992). Auditory selective attention in middle-aged and elderly subjects: An event-related brain potential study. Electroencephalogr. Clin. Neurophysiol. Potentials Sect..

[50] Müller BW, Achenbach C, Oades RD, Bender S, Schall U (2002). Modulation of mismatch negativity by stimulus deviance and modality of attention. NeuroReport.

[51] Patel SH, Azzam PN (2005). Characterization of N200 and P300: Selected studies of the event-related potential. Int. J. Med. Sci..

[52] Tarkka IM, Stokić DS, Basile LFH, Papanicolaou AC (1995). Electric source localization of the auditory P300 agrees with magnetic source localization. Electroencephalogr. Clin. Neurophysiol. Potentials Sect..

[53] Donchin E, Coles MGH (1988). Is the P300 component a manifestation of context updating?. Behav. Brain. Sci..

[54] Polich J (2004). Clinical application of the P300 event-related brain potential. Phys. Med. Rehabil. Clin. N. Am..

[55] Polich J (2007). Updating P300: An integrative theory of P3a and P3b. Clin. Neurophysiol..

[56] Bekinschtein TA, Dehaene S, Rohaut B, Tadel F, Cohen L, Naccache L (2009). Neural signature of the conscious processing of auditory regularities. Proc. Natl. Acad. Sci. U. S. A..

[57] Haroush K, Hochstein S, Deouell LY (2010). Momentary fluctuations in allocation of attention: Cross-modal effects of visual task load on auditory discrimination. J. Cogn. Neurosci..

[58] Sussman E, Kujala T, Halmetoja J, Lyytinen H, Alku P, Näätänen R (2004). Automatic and controlled processing of acoustic and phonetic contrasts. Hear. Res..

[59] Lang AH (1995). Practical issues in the clinical application of mismatch negativity. Ear Hear..

[60] Sallinen M, Lyytinen H (1997). Mismatch negativity during objective and subjective sleepiness. Psychophysiology.

[61] Gomes H, Molholm S, Ritter W, Kurtzberg D, Cowan N, Vaughan HG (2000). Mismatch negativity in children and adults, and effects of an attended task. Psychophysiology.

[62] Tervaniemi, .M, Maury, S., & Näätänen, R. Neural representations of abstract stimulus features in the human brain as reflected by the mismatch negativity. *Neuroreport,* **5,** 844–846 (1994). 10.1097/00001756-199403000-00027 8018861

[63] Carral V, Corral M-J, Escera C (2005). Auditory event-related potentials as a function of abstract change magnitude. NeuroReport.

